# Building a diagnostic prediction model for severe *Mycoplasma pneumoniae* pneumonia in children using machine learning

**DOI:** 10.3389/fpubh.2025.1585042

**Published:** 2025-09-18

**Authors:** Chuxiong Gong, Helang Yue, Qinhong Li, Yanfei Yang, Hongyan Li, Tingting Hao, Hongrui Wu, Yanwei Xu, Qiyin Huang, Xingzhu Liu, Yuqin Wu

**Affiliations:** ^1^Department of Cardiology, Kunming Children’s Hospital, Kunming, Yunnan, China; ^2^Kunming Medical University, Kunming, Yunnan, China; ^3^Department of Special Needs Ward, Kunming Children’s Hospital, Kunming, Yunnan, China; ^4^Medical College of Dali University, Dali, Yunnan, China

**Keywords:** *Mycoplasma pneumoniae* pneumonia, severe *Mycoplasma pneumoniae* pneumonia, LASSO, random forest, predictive model

## Abstract

**Objective:**

*Mycoplasma pneumoniae* is the leading pathogen of community-acquired pneumonia in children. In recent years, *M. pneumoniae* pneumonia (MPP) has shown a global pandemic trend. The increasing incidence of severe MPP (SMPP) leads to complications and even deaths, severely impacting prognosis and quality of life. Our study aimed to use machine learning to construct an early diagnostic model for severe MPP in children. It supports early prediction, prevention, and individualized precise treatment of SMPP.

**Methods:**

We collected medical records from 372 MPP cases. We compared case characteristics between groups with and without SMPP and used a random forest to screen key factors. We then constructed a multivariate logistic prediction model. We evaluated the model with ROC curves, calibration curves, and DCA. Five-fold cross-validation tested prediction stability.

**Results:**

We identified ESR, PCT, IL-6, and lung auscultation as key factors to construct the prediction model. The model’s ROC was 0.964 (95% CI: 0.945–0.983). Calibration curves and DCA confirmed model accuracy. Five-fold cross-validation validated internal stability.

**Conclusion:**

Our study developed a prediction model with good efficacy for early SMPP risk assessment. Our research provides a basis for clinical early prediction and prevention of SMPP, reducing its risk and offering a foundation for individualized treatment and improved long-term outcomes in affected children.

## Introduction

*Mycoplasma pneumoniae* (MP) is a pathogenic microorganism situated between bacteria and viruses, primarily targeting the human respiratory tract. It is one of the most common pathogens of community-acquired pneumonia (CAP) in children (1). *M. pneumoniae* pneumonia (MPP), induced by MP, accounted for 40% of acute inflammatory cases in pediatric CAP (2, 3), with 18% of affected children requiring hospitalization. The clinical manifestations of MPP varied, including pulmonary rales, shortness of breath, and cough, with some patients experiencing wheezing. In recent years, MPP showed a widespread trend, with a decreasing age of onset. Notably, since 2000, macrolide antibiotic resistance rates rapidly increased worldwide (reaching 69 to 95% in China), leading to more cases of severe *M. pneumoniae* pneumonia (SMPP) (4, 5). Moreover, children with SMPP often suffered from pulmonary complications and extrapulmonary organ damage, such as pleural effusion, necrotizing pneumonia, myocarditis, and vascular embolism, risking multiple organ failure and even death, posing a serious threat to children’s health (5, 6). Recently, the mortality rate of SMPP gradually rose, with a rate of approximately 0.1–1% (6). Additionally, severe sequelae such as pulmonary fibrosis (PF), bronchiolitis obliterans, and unilateral hyperlucent lung increasingly affected long-term prognosis and quality of life in children. Therefore, early prediction of SMPP occurrence in clinical settings was crucial for implementing timely and effective targeted interventions (7).

Currently, there was no unified understanding of the clinical manifestations, pathogenesis, laboratory diagnostic methods and indicators, and pulmonary imaging changes of pediatric MPP and SMPP (8, 9). Reliable tools or indicators for the early prediction of SMPP infection were also lacking to guide clinical prevention and treatment. Hence, our research aimed to utilize machine learning to construct an early diagnostic prediction model for severe *Mycoplasma pneumoniae* pneumonia in children. This model would facilitate early identification of severe cases, enabling precise medical intervention, reducing complications, and shortening the average hospital stay.

## Materials and methods

### Participants

Our study retrospectively collected cases of children diagnosed with Community-Acquired Pneumonia (CAP) and related clinical data from July 2023 to July 2024 at a tertiary hospital. All diagnoses of Mycoplasma pneumonia were confirmed based on positive nucleic acid tests for *M. pneumoniae*. The collection of case data received ethical approval from the Ethics Committee of Kunming Children’s Hospital.

### Diagnostic criteria

The diagnosis of MPP: Confirmation is achieved by fulfilling at least one of the following laboratory diagnostic criteria: a four-fold or greater increase in MP antibody titer during the recovery phase compared to the acute phase, or a positive MP culture or MP-DNA/RNA detection. Refractory MPP is defined as patients meeting the aforementioned MPP criteria who continue to exhibit persistent fever and worsening lung imaging findings despite receiving standard treatment with macrolides for seven or more days. Severe MPP (SMPP) is diagnosed in patients who meet the established MPP criteria and also fulfill the criteria for severe pneumonia as outlined in the “Guideline for the management of community-acquired pneumonia in children (2023)”.

The diagnosis of SMPP (10): (1) continuous high fever (above 39 °C) for ≥5 days or fever for ≥7 days; (2) development of wheezing, shortness of breath, dyspnea, chest pain, or hemoptysis; (3) the presence of extrapulmonary complications; (4) pulse oxygen saturation ≤0.93 at rest, breathing room air; (5) imaging findings characterized by at least one of the following: uniform and consistent high-density consolidation of ≥2/3 of a single lobe, high-density consolidation of two or more lobes with a moderate to large pleural effusion or with localized bronchitis, diffuse capillary bronchitis in one lung, or capillary bronchitis of ≥4/5 lobes in both lungs, combined with bronchitis, and atelectasis resulting from the formation of mucous emboli; (6) progressively aggravated clinical symptoms, with extension of the lesion range by more than 50% in 24–48 h based on imaging; or (7) an obvious increase in C-reactive protein (CRP), lactate dehydrogenase (LDH), or D-dimer levels. Patients with immunodeficiency and those taking immunosuppressants were excluded.

### Inclusion and exclusion criteria

Inclusion criteria: (1) Complete clinical data for all children; (2) No treatment prior to laboratory tests. Exclusion criteria: (1) Presence of underlying conditions such as lung malformations, pulmonary vascular anomalies, congenital heart disease, hematological diseases, immune system disorders, and endocrine genetic metabolic diseases; (2) History of *Mycoplasma pneumoniae* (MP) infection within 1 year; (3) History of severe pneumonia within 1 year. (4) History of other infections within 1 month, including but not limited to: respiratory infections, gastrointestinal infections, and neurological infections.

### Included factors

We included 19 relevant factors. Basic demographic information included age, gender, and residence of the children. Laboratory indicators included Erythrocyte Sedimentation Rate (ESR), Procalcitonin (PCT), Interleukin 6 (IL6), White Blood Cell count (WBC), Platelet count (PLT), C-Reactive Protein (CRP), Immunoglobulin G (IgG), Immunoglobulin M (IgM), Immunoglobulin A (IgA), Complement component 4 (C4), and Complement component 3 (C3). Additionally, we assessed Lung Auscultation, the number of Fever Days, Feeding Method, Gestational Age, and Delivery method to better understand their relationships in our research context.

### Model development

First, we divided all cases into two groups: the SMPP group and the non-SMPP group, based on the presence of SMPP. We compared 19 relevant factors between these groups and included statistically significant ones in further analysis. We then used the “glmnet” R package to conduct Least Absolute Shrinkage and Selection Operator (LASSO) analysis to identify more important factors for outcome prediction. LASSO is a regularization method for linear regression that adds an L1 penalty, facilitating variable selection and model simplification. Its main advantage was reducing the coefficients of unimportant variables to zero, retaining only key variables and reducing model complexity to prevent overfitting. We employed 5-fold cross-validation to select the optimal lambda value, either lambda.min or lambda.1se, where a larger lambda indicated higher regularization strength and resulted in fewer selected variables. We filtered variables based on optimal and maximum lambda values. Then, to identify predictors more related to outcomes, we applied the random forest algorithm from the “randomForest” R package to further filter indicators selected by LASSO analysis and measured the impact of each predictor on model performance using the Increase in Mean Squared Error (IncMSE). To prevent overfitting, we performed 5-fold cross-validation on the random forest model to determine the optimal K value and conducted random forest computation, assessing the fit using *R*^2^. We then computed the correlation among key variables selected by the random forest using the “corrplot” R package to avoid collinearity bias due to high correlations. Finally, we used the “rmda” R package to include the final selected factors in a multivariable logistic regression for model development and created a nomogram of the optimal model using the “rms” R package.

### Model evaluation and validation

To rigorously evaluate the performance and generalizability of our predictive model, we employed a multifaceted statistical approach. We initiated our analysis by generating Receiver Operating Characteristic (ROC) curves using the “pROC” R package pROC: an R package for ROC and AUC computation and visualization, calculating the Area Under the Curve (AUC) to quantify the model’s ability to discriminate between different outcomes. Next, we assessed model calibration, ensuring that predicted probabilities aligned with observed frequencies, by creating a calibration curve within the “ResourceSelection” R package. We then conducted Decision Curve Analysis (DCA) using the decision_curve function to assess the model’s clinical utility, evaluating its net benefit across different risk thresholds. Furthermore, to establish the robustness of our model and assess its performance on independent datasets, we implemented five-fold cross-validation. This process involved partitioning the dataset into five equally sized folds using the ‘createFolds’ function from the ‘caret’ R package. For each fold, the model was trained on the data from the other four folds and then validated on the held-out data. Within each iteration, we calculated the AUC and assessed the calibration. This process was repeated five times, with each fold serving as the validation set once. The average AUC across the five folds provides a reliable estimate of the model’s expected performance, and the variability across folds indicates the model’s stability. This approach ensures a more reliable assessment of the model’s predictive power and generalizability.

### Statistical analysis

All statistical analyses were conducted using R version 4.4.1. Normally distributed continuous data were expressed as mean ± standard deviation (mean ± SD) and compared between two groups using the independent samples *t*-test. Non-normally distributed data were described using quartiles and compared with non-parametric rank sum tests. Categorical data were expressed as proportions and compared between groups using the chi-square test. A *p*-value < 0.05 was considered statistically significant.

## Results

### Description and comparison of clinical characteristics between two groups

Among the enrolled patients, there were 83 cases (22.31%) in the SMPP group and 289 cases (77.69%) in the no-SMPP group. Comparing the clinical data of the two groups, we found significant statistical differences in maternal age, ESR, WBC, VitD, IgM, IgA, C3, PCT, IL6, CRP, IgG, gestational age, sex, and lung auscultation (Table 1).

**Table 1:** Comparison of clinical characteristics between two groups.

Factor	SMPP (*n* = 83)	No-SMPP (*n* = 249)	*p*-value
Age	1498.12 ± 870.2	1148.69 ± 866.21	0.002
ESR	41.3 ± 16.95	20.85 ± 15.58	<0.001
WBC	13.68 ± 6.56	11.22 ± 6.17	0.003
PLT	307.75 ± 116.34	305.02 ± 104.97	0.85
VitD	40.45 ± 14.4	45.23 ± 16.75	0.013
IgM	0.85 ± 0.44	0.99 ± 0.51	0.022
IgA	0.53 ± 0.37	0.63 ± 0.42	0.043
C3	0.96 ± 0.26	1.05 ± 0.3	0.013
PCT	3.19(0.25–12.14)	0.25(0.25–0.34)	<0.001
IL6	40.35(25.55–63.89)	13.92(6.29–21.65)	<0.001
CRP	20.4(12.45–33.23)	6.29(0.5–21.86)	<0.001
IgG	6.18(4.62–7.39)	6.69(5.2–8.3)	0.024
C4	0.24(0.16–0.31)	0.25(0.18–0.33)	0.145
Gestational_age	0(0–1)	0(0–1)	0.029
Sex			0.009
Female	31(37.35)	136(54.62)	
Male	52(62.65)	113(45.38)	
Residence			0.39
No	26(31.33)	93(37.35)	
Yes	57(68.67)	156(62.65)	
Lung auscultation			<0.001
No	4(4.82)	159(63.86)	
Yes	79(95.18)	90(36.14)	
Feeding method			0.803
No	45(54.22)	138(55.42)	
Yes	38(45.78)	111(44.58)	
Delivery			0.791
No	52(62.65)	162(65.06)	
Yes	31(37.35)	87(34.94)	

### Key factor selection

**Figure 1:**
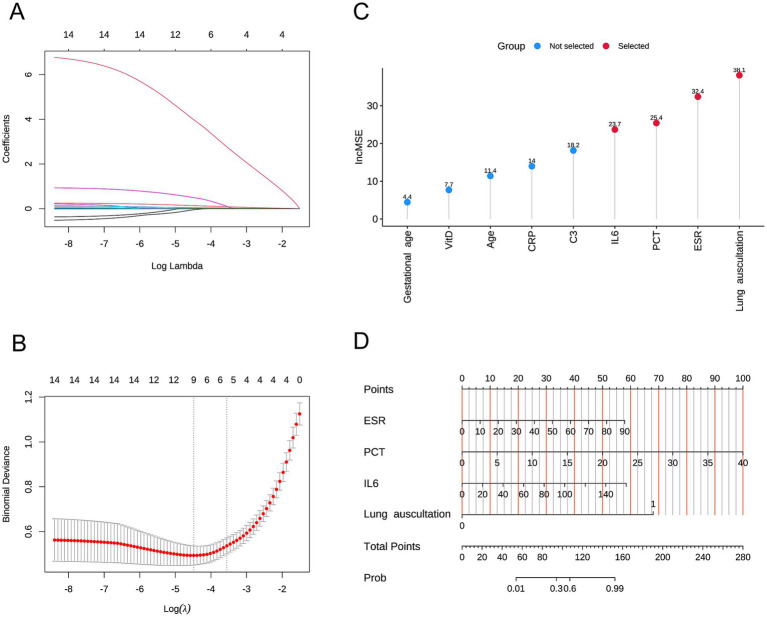
**(A,B)** Variables selected by Lasso analysis. **(C)** Nomogram of the multivariable logistic regression model. **(D)** Nomogram of the predictive model.

We included these 14 factors in LASSO regression to select those with greater predictive value. We used five-fold cross-validation to choose the optimal lambda value of 0.01128. The factors with the best linear relationship, namely age, ESR, VitD, C3, PCT, IL6, CRP, gestational age, and lung auscultation, were selected for further analysis using random forests (Figures 1A,B). Before applying the random forest algorithm, we used five-fold cross-validation to find the optimal K value of 450. We ranked the nine factors by IncMSE from the random forest analysis and found that ESR, PCT, IL6, and lung auscultation had significantly higher IncMSE values than the other factors (Figure 1C). Therefore, we included these four factors in the subsequent model construction. Additionally, the evaluation of the random forest algorithm showed an R-squared of 0.939 and a root mean squared error of 0.107, indicating a good model fit and strong predictive ability.

### Development and evaluation of the predictive model

**Figure 2:**
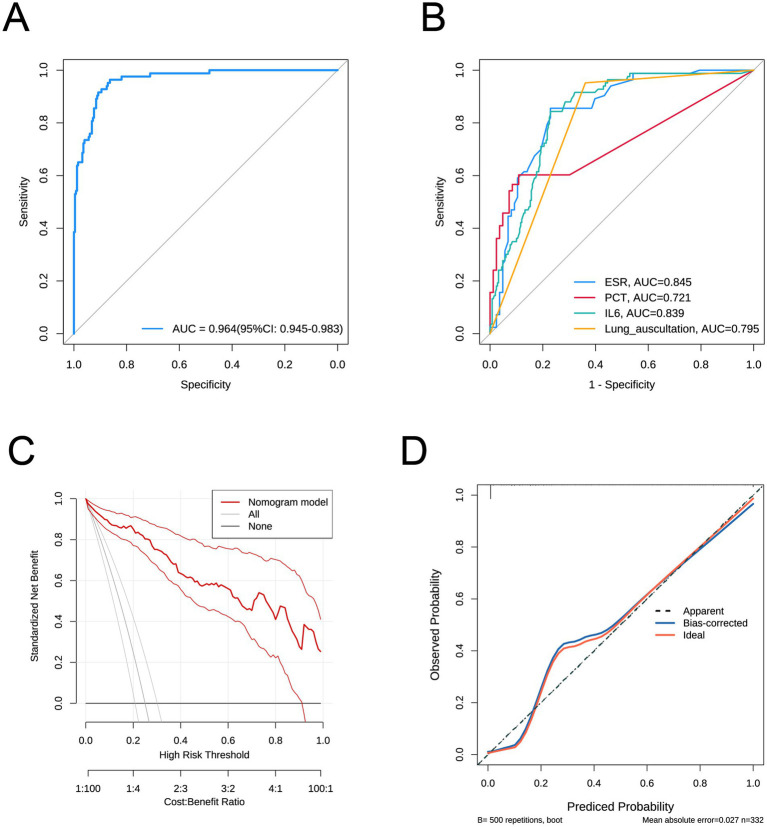
**(A)** ROC curve of the predictive model. **(B)** ROC curves for individual factors ESR, PCT, IL6, and lung auscultation. **(C)** Calibration curve of the predictive model. **(D)** curve of the predictive model.

Addressing Reviewer Comment 3 concerning the insufficient description of our predictive model, specifically regarding the absence of detailed parameters (OR, 95% CI, P, and Coefficients) in the forest plot, we have significantly revised our manuscript to offer a more comprehensive presentation. Following established methodology, a multifactor logistic regression model was constructed. To address this, our model identified elevated ESR, PCT, IL6, and a positive lung auscultation as statistically significant independent risk factors. These four variables were then incorporated, which include: ESR (coefficient = 0.06, *p* = 0.001, OR = 1.062 [95% CI: 1.026–1.099]), PCT (coefficient = 0.233, *p* < 0.001, OR = 1.263 [95% CI: 1.116–1.429]), IL6 (coefficient = 0.034, *p* < 0.001, OR = 1.035 [95% CI: 1.015–1.055]), and positive lung auscultation (coefficient = 6.346, *p* < 0.001, OR = 570.265 [95% CI: 53.912–6032.056]). The forest plot clearly displays the independent risk associated with the four variables. Furthermore, we have enhanced the integration of tables, figures, and accompanying text. The model’s performance, including its high AUC of 0.964 (95% CI: 0.945–0.983) in Figure 2A, its superiority over individual predictors (Figure 2B), robust cross-validation results, and well-calibrated performance as demonstrated via the calibration and DCA curve (Figures 2C,D), validates the model’s reliability, thereby enriching the overall clarity of the data. Moreover, we have also enhanced the “Methods” and “Results” sections to explicitly link variable selection to the derived model. These thorough revisions fully address the reviewer’s comments, strengthening the clarity, transparency, and comprehensiveness of our manuscript.

## Discussion

The MP was the primary pathogen for pediatric CAP. In recent years, MPP showed a global epidemic trend. Particularly, an increasing number of SMPP cases occurred, severely affecting children’s prognosis and quality of life. Therefore, our study analyzed the clinical data of 372 children with MPP and compared clinical characteristics between those who developed SMPP and those who did not (9). We used Lasso analysis and random forest methods to identify ESR, PCT, IL-6, and lung auscultation as key factors. These factors were used to construct a multivariate logistic prediction model. The ROC curve demonstrated good predictive capability of the model. Finally, we then performed five-fold cross-validation to verify the model’s predictive stability.

Clinically, early recognition of SMPP and severe MPP is crucial for timely targeted interventions. Early warning indicators include the host’s baseline physical condition, clinical symptoms, laboratory markers, and treatment response. Excessive immune-inflammatory responses play critical roles in MPP infection and its pathogenesis, prompting increased research into MPP cytokine profiles as a research focus (11, 12). These studies aim to determine whether cytokines can serve as reliable biomarkers to predict MPP severity, providing clinicians with early, precise guidance for individualized treatment plans. Numerous studies have reported inflammatory cytokines such as IL and chemokines play important roles in assessing and determining the severity of pediatric MPP (13–15). However, discrepancies exist among research conclusions.

The IL are small proteins secreted by immune cells. They respond to inflammatory signals, regulate immune cell function, and modulate the intensity and direction of the immune response (16, 17). IL levels are key initiators and regulators of the acute-phase response, playing a crucial role in immune and inflammatory reactions (18, 19). MP infection induces a strong inflammatory response in the host, leading to the secretion of numerous cytokines. IL-6, an important pro-inflammatory cytokine, is significantly elevated in patients (20). Elevated IL-6 reflects the intensity of the body’s inflammatory response to MP infection and correlates with the degree of inflammatory infiltration and damage to the lung tissue. In severe MPP, the sustained high levels of IL-6 may indicate uncontrolled inflammation, leading to increased lung damage and even a “cytokine storm”-like reaction, causing systemic inflammatory response syndrome. Therefore, IL-6 is considered a potential biomarker for assessing the severity and prognosis of MPP (16). However, IL-6 is also influenced by various factors, such as glucocorticoid therapy, which may suppress IL-6 production, leading to a decrease in its levels. This, however, does not necessarily mean the infection has completely resolved; it may simply indicate that the inflammatory response has been suppressed. ESR is an important inflammatory marker for assessing the severity of infection. During pathogen infections, acute-phase reactants such as fibrinogen, CRP and haptoglobin increase, promoting the formation of rouleaux (stacking of red blood cells), which leads to an accelerated ESR 2–3 days after the onset of inflammation (21). In MP, especially severe ones, the body activates the immune system to produce inflammatory mediators and the aforementioned acute-phase proteins (such as the elevated fibrinogen, CRP, and immunoglobulins mentioned in the text). These changes cause the ESR to accelerate 2–3 days after inflammation begins and remain elevated during the active phase of the disease (22). High ESR levels indicate a persistent and strong inflammatory response within the body, which is consistent with the pathophysiology of MPP (23). In patients with severe MPP, the ESR further accelerates, reflecting more extensive tissue damage and a more intense inflammatory response. Although ESR is affected by non-specific factors (such as anemia, hypercholesterolemia, pregnancy, age, and sex), as a simple and readily available test, it still effectively reflects the inflammatory status and severity of MPP (8). PCT is an important biomarker for bacterial infections, especially severe bacterial infections, and is widely used clinically to differentiate between bacterial and viral infections, as well as to assess the severity and prognosis of infection (24). *M. pneumoniae* pneumonia itself is an atypical pathogen infection, and PCT levels usually do not significantly increase initially. However, as mentioned in the text, MPP often coexists with secondary bacterial infections during its progression, or the MP infection itself can also induce a strong inflammatory response (25). PCT levels will significantly increase when MPP is complicated by a bacterial infection or when MP infection leads to severe systemic inflammatory response syndrome. The nature and extent of lung rales are important clinical indicators for assessing lung inflammation and the extent of airway involvement (26). MP infection often causes bronchiolitis changes, leading to bronchial wall edema, spasm, and intraluminal mucus plugs formation, which may lead to airway narrowing, thereby producing dry rales, especially wheezing. Persistent or extensive dry rales indicate a more severe degree of airway obstruction, which may be related to lower airway hyperreactivity or airway remodeling caused by MPP, and is associated with complications.

This study has limitations due to a small sample size and limited indicators, leading to potential confounding factors, restricting exploration such as subgroup analysis by gender to explore differences among subgroups; relationships between quantitative indicators and diverse clinical manifestations; changes in these indicators between acute and recovery phases. Future clinical practice should incorporate more samples and indicators to establish a simpler, stable diagnostic model and more cost-effective treatment plans. Additionally, we aim to conduct multi-omic studies, including radiomics, proteomics, metabolomics, and genomics, to enrich SMPP predictive biomarkers and refine diagnostic models.

In conclusion, our research developed a predictive model with good performance using machine learning to assess severe MPP risk in children in China. This model was validated across subgroups based on disease stage, gender, and residence. Our study provides evidence for early SMPP identification, enabling early preventive measures and reducing SMPP risk, laying a theoretical foundation for precision medicine.

## Conclusion

Our study developed a prediction model with good efficacy for early SMPP risk assessment. The model was effectively validated across gender, age stages, vitamin D levels, and other factors. Our research provides a basis for clinical early prediction and prevention of SMPP, reducing its risk and offering a foundation for individualized treatment and improved long-term outcomes in affected children.

## Data Availability

The original contributions presented in the study are included in the article/supplementary material, further inquiries can be directed to the corresponding authors.
