# Supplementary figures and images for: Building a diagnostic prediction model for severe Mycoplasma pneumoniae pneumonia in children using machine learning
Source: Front Public Health. 2025 Sep 18;13:1585042. doi: 10.3389/fpubh.2025.1585042 (PMC12488690; doi:10.3389/fpubh.2025.1585042)

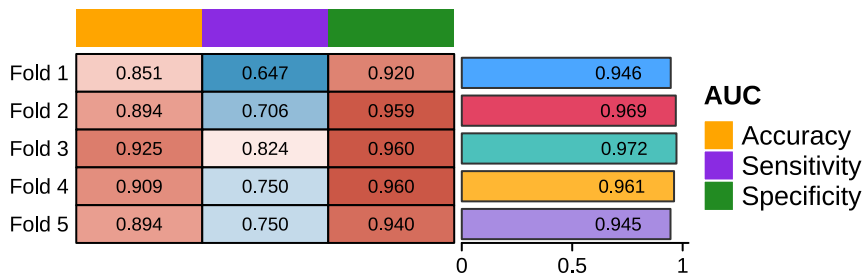

Supplement: Supplementary file 1 [file Table_1.pdf]
